# Supplementary material for: Safety, Efficacy and Long-Term Outcomes of Patients Treated with the Occlutech Paravalvular Leak Device for Significant Paravalvular Regurgitation
Source: J Clin Med. 2022 Apr 1;11(7):1978. doi: 10.3390/jcm11071978 (PMC8999580; doi:10.3390/jcm11071978)
Supplement: Supplementary file 1 [file jcm-11-01978-s001.zip › jcm-1613465-supplementary material.pdf]

Supplementary Material

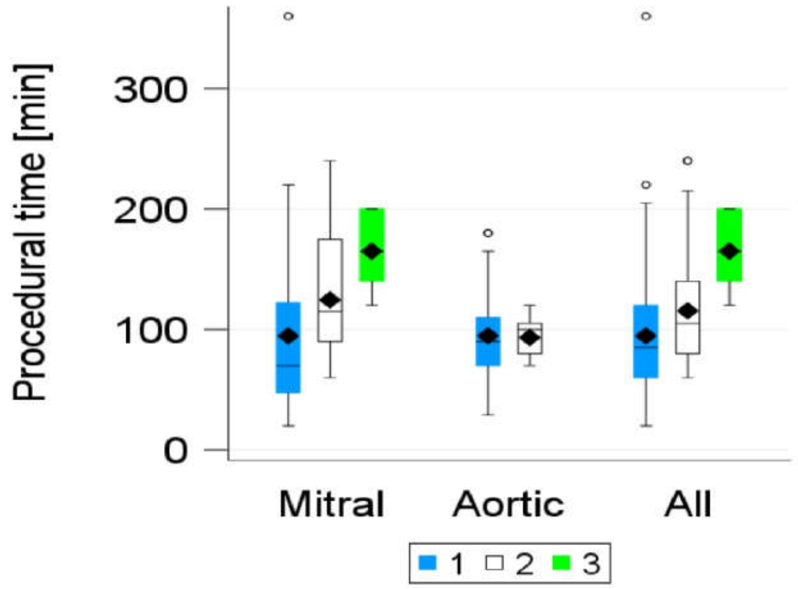

Figure S1. Procedural time [min] per Number of leaks by Stratum (SAF). SAF: safety analysis set.

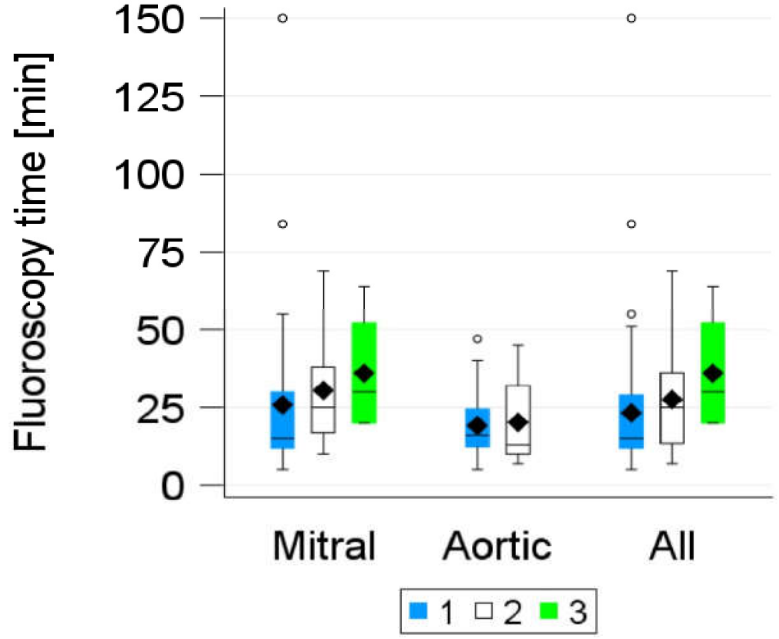

Figure S2. Fluoroscopy time [min] per Number of leaks by Stratum (SAF). SAF: safety analysis set.

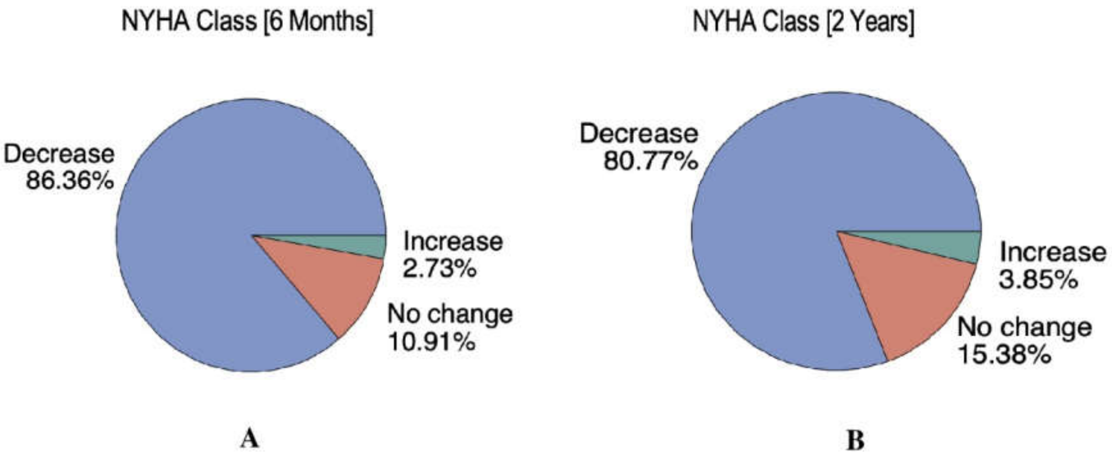

Figure S3. Qualitative change for NYHA Class BL vs 6M, FAS (**A**) and BL vs 2Y, FAS (**B**). BL: baseline; 6M: 6 months; FAS: full analysis set.

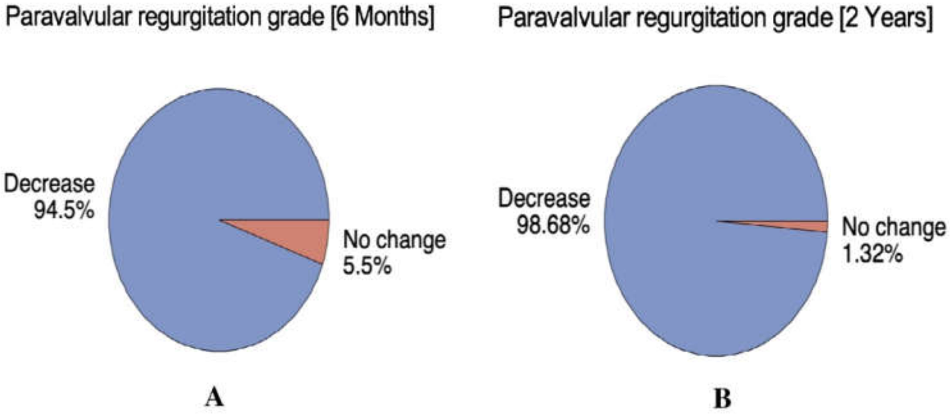

Figure S4. Qualitative change (to Base) for paravalvular regurgitation grade BL vs 6M, FAS (**A**) and BL vs 2Y, FAS (**B**). BL: baseline; 6M: 6 months; FAS: full analysis set.

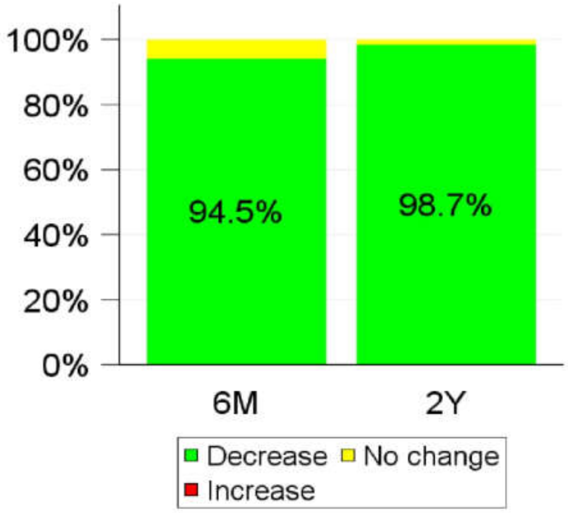

Figure S5. Qualitative change (to Base) for Paravalvular regurgitation grade BL vs 6M and 2 Y. BL: baseline; 6M: 6 months; FAS: full analysis set.

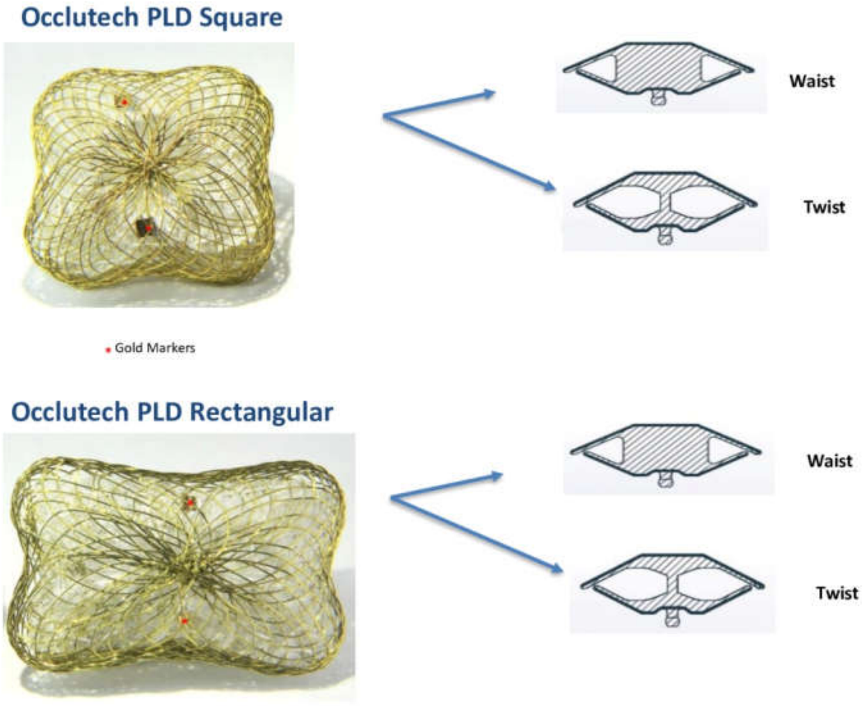

Figure S6. The Occlutech ® Paravalvular Leak Device (PLD).

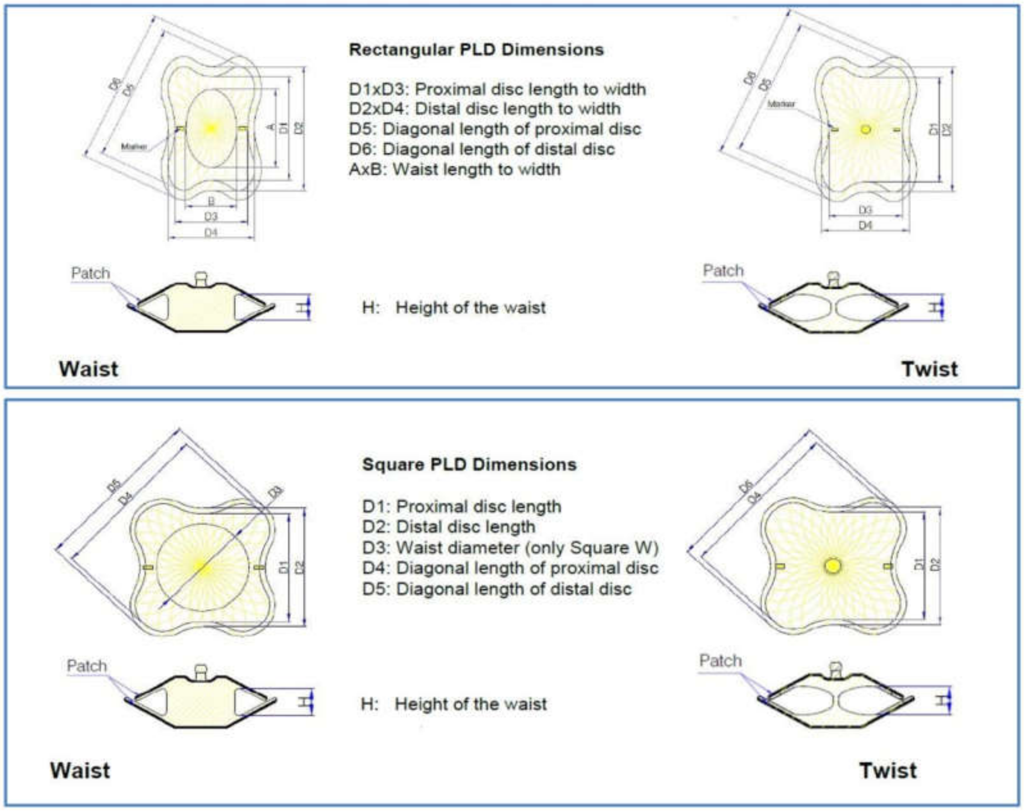

Figure S7. Technical drawings showing Rectangular and Square PDL dimensions for the two types of connecting, *waist* and *twist*.

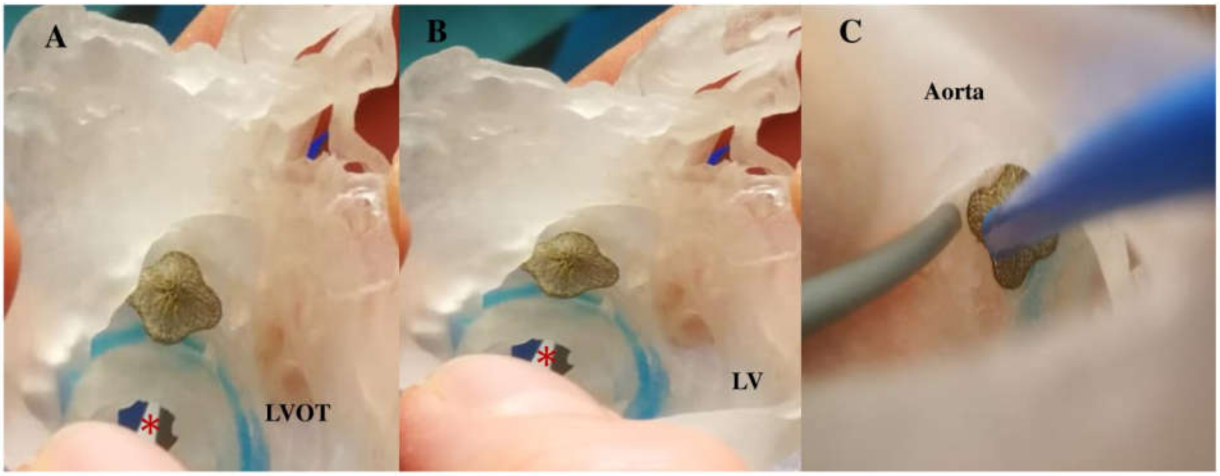

Figure S8. 3D patient-specific simulation-guided treatment strategy.
